# The prognostic value of patient-reported outcomes in allogeneic hematopoietic stem cell transplantation: exploratory analysis of a randomized nutrition intervention trial

**DOI:** 10.1007/s00277-023-05149-x

**Published:** 2023-03-03

**Authors:** Anne Marte Gudmundstuen, Fabio Efficace, Geir Erland Tjønnfjord, Kristin Joan Skaarud, Francesco Cottone, Marianne Jensen Hjermstad, Per Ole Iversen

**Affiliations:** 1 Department of Haematology, Oslo University Hospital, 4950 Nydalen, NO-0424 Oslo, Norway; 2 Department of Nutrition, Institute of Basic Medical Science, University of Oslo, Oslo, Norway; 3 Italian Group for Adult Hematologic Diseases (GIMEMA), Data Centre and Health Outcomes Research Unit, Rome, Italy; 4 K.G. Jebsen Centre for B Cell Malignancies, University of Oslo, Oslo, Norway; 5 Institute of Clinical Medicine, University of Oslo, Oslo, Norway; 6 Lovisenberg Diaconal University College, Oslo, Norway; 7 European Palliative Care Research Centre (PRC) Department of Oncology Oslo University Hospital/Institute of Clinical Medicine, University of Oslo, Oslo, Norway; 8 Regional Advisory Unit for Palliative Care, Department of Oncology, Oslo University Hospital, Oslo, Norway

**Keywords:** Allogeneic stem cell transplantation, Patient-reported outcomes, EORTC QLQ-C30, Overall survival

## Abstract

Whether patient-reported outcomes (PROs) can predict overall survival (OS) and non-relapse mortality (NRM) among recipients of allogeneic stem cell transplantation (allo-HSCT), is unclear. We performed an exploratory analysis of the prognostic value of patient-reported outcomes (PROs) among 117 recipients of allogeneic stem cell transplantation (allo-HSCT) who participated in a randomized nutrition intervention trial. Cox proportional hazards models were used to investigate possible associations between PROs collected pre-allo-HSCT (baseline) using scores from the EORTC Quality of Life Questionnaire-Core 30 (QLQ-C30) and 1-year overall survival (OS), whereas logistic regression was used to study associations between these PROs and 1-year non-relapse mortality (NRM). Multivariable analyses indicated that only the Hematopoietic Cell Transplantation Comorbidity Index (HCT-CI) and the European Bone Marrow Transplantation (EBMT) risk score were associated with 1-year OS. In the multivariable model including clinical-sociodemographic factors for 1-year NRM, our analysis showed that living alone (*p*=0.009), HCT-CI (*p*=0.016), EBMT risk score (*p*=0.002), and stem cell source (*p*=0.046) could be associated with 1-year NRM. Moreover, in the multivariable model, our analysis showed that only appetite loss from the QLQ-C30 was associated with 1-year NRM (*p=*0.026). In conclusion, in this specific setting, our analysis suggests that the commonly used HCT-CI and EBMT risk scores could be predictive for both 1-year OS and 1-year NRM, whereas baseline PROs in general were not.

**Supplementary Information:**

The online version contains supplementary material available at 10.1007/s00277-023-05149-x.

## Introduction

Patients undergoing allogeneic hematopoietic stem cell transplantation (allo-HSCT) have a high risk of transplant-related complications such as toxicity, infections, and graft-versus-host disease (GVHD). Approximately 50% of allo-HSCT recipients become long-term survivors [1, 2]. However, the treatment is associated with significant morbidity due to acute and late complications and long-lasting side effects resulting in a high symptom burden with fatigue, impaired physical function, reduced quality of life (QoL), poor sleep, and reduced appetite persisting for several months after the transplantation [3, 4].

To predict mortality and transplant-related side effects that informs individually adapted clinical care, patients are scored according to standard clinical prognostic scoring systems prior to allo-HSCT, such as the Hematopoietic Cell Transplantation Comorbidity Index (HCT-CI) and the European Bone Marrow Transplantation (EBMT) risk score. However, these scoring systems do not include patient-reported outcomes (PROs), which not only provide important information for clinical care, but may also predict survival [5]. For example, PROs can capture the unique patient perspective on the burden of disease and treatment and guide tailored interventions. There is now empirical evidence indicating that PROs, such as symptoms or functional aspects, provide independent prognostic information on survival in several cancer populations [6–10]. In line with this, studies show that patient-reported physical functioning is a frequent prognostic factor for overall survival (OS) independent of other known traditional prognostic indicators [9, 10].

However, less evidence is available on the potential prognostic value of PRO data in patients undergoing allo-HSCT. Hamilton et al. showed that pre-transplant QoL and physical well-being were associated with reduced risk of overall mortality after allo-HSCT [11]. Also, Wood et al. found that lower pre-transplant physical QoL scores were predictive of survival and transplant-related mortality among allo-HSCT patients [12]. Moreover, Palmer et al. found that changes in PROs predicted survival in patients with chronic GVHD [13]. Some studies have also shown that inclusion of PROs (i.e., fatigue) in well-established risk score classifications may improve their prognostic accuracy, as was the case for patients with myelodysplastic syndromes [14, 15].

If, as studies above indicate, PROs are documented to have a prognostic value, it can be relevant to include PROs more systematically in clinical care and follow-up. This could in turn help guide interventions to reduce mortality and morbidity, as well as improving QoL after allo-HSCT.

Because of the scarcity of information on the prognostic value of PROs in this group of patients, we conducted a secondary analysis of data from an open randomized controlled trial (RCT) examining a nutrition intervention in patients undergoing allo-HSCT following myeloablative conditioning [16, 17]. The main aim of the current study was to assess through an exploratory analysis the possible prognostic value of baseline PROs while also considering other key sociodemographic and clinical factors, for 1-year OS and for 1-year non-relapse mortality (NRM) after allo-HSCT. We also examined whether baseline PROs could add value to the currently used scoring systems (HCT-CI and EBMT risk score).

## Patients and methods

### Approvals

The original RCT and the current study were approved by the Regional Committee for Medical and Health Research Ethics South East Norway (#S-09136c 2009/2115), and the Data Protection Supervisor, Oslo University Hospital and conducted in accordance with the Declaration of Helsinki. All patients gave written informed consent. The original RCT is registered in ClinicalTrials.gov, ID NCT01181076.

### Study design and patient selection in the RCT

One hundred and seventeen patients (intervention *n*=57, control *n*=60) were included in this RCT conducted at the Department of Haematology, Oslo University Hospital from August 2010 to February 2017. The aims of the RCT were to assess the impact of optimized energy and protein intake compared to routine hospital nutrition support on global QoL and clinical outcomes three months after allo-HSCT. A detailed description of the RCT and main clinical outcomes have been reported previously and showed no significant differences among the two study groups on any of the QoL-C30 scales or items [17].

### Nutrition intervention in the original randomized controlled trial

In short, the nutritional intervention started upon hospital admission with optimization of energy and protein intake until discharge (usually after 3–5 weeks) [16]. The patients in the intervention group had their daily energy and protein requirement estimated according to World Health Organization recommendations, i.e., 126 to 167 kJ (30 to 40 kcal) per kg each day and 1.5–2.0 g protein/kg/each day [18] and validated by measuring the patients’ energy expenditure with indirect calorimetry, adding an activity factor. Oral intake was monitored by the patient’s self-reports with additional enteral parenteral nutrition if the estimated intake was insufficient, i.e,. lower than the estimated energy needs. Patients in the control group received a standard amount of parenteral nutrition combined with oral intake if possible.

### Sociodemographic and clinical factors

At enrollment, all the participants provided information on the following sociodemographic factors: age, sex, education, and marital status. The electronic health records were used to provide information on disease-, transplant- and treatment-specific information such as diagnosis, conditioning regime, admission dates, duration of hospitalization, days alive, and duration of out of the hospital within the first year after allo-HSCT.

### Patient-reported outcomes assessment

The European Organisation for Research and Treatment of Cancer Core Quality of Life Questionnaire (QLQ-C30) was used to assess QoL at baseline (i.e., on day 8 or 7 before allo-HSCT) [19]. The QLQ-C30 includes five functional scales (role, physical, cognitive, emotional and social), three symptom scales (nausea/vomiting, fatigue, and pain), and six single items (insomnia, dyspnea, appetite loss, constipation, diarrhea, and financial problems). This questionnaire also includes a global health status/quality of life (QoL) scale. All scales and single items are transformed into standardized scores ranging from 0 to 100, with higher scores for the functioning scales and global health status/QoL scale indicating better outcomes, while higher scores on the symptom scales and single items indicate greater symptom severity [19].

### Clinical scoring systems

Clinical- and transplant-related data were registered daily during hospitalization and later retrieved from the medical health records. This included diagnosis and progression of disease, conditioning regime, donor information, stem cell source, the HCT-CI, and EBMT scores. The HCT-CI score summarizes the presence of 17 comorbidities, and as such, does not denote the patient perspective [20]. Patients are classified in three risk groups based on the sum score (low risk = 0 points, intermediate risk =1-2 points, or high risk > 3 or more points), which correlates highly with 2-year NRM [21]. The EBMT risk score includes five factors: stage of the disease, age of the patient, time from diagnosis, donor type, and donor-recipient sex combination. This risk score complements the HCT-CI classification by emphasizing transplant-related factors [22]. In addition, the Eastern Cooperative Oncology Group (ECOG) score for assessment of physical performance was used at baseline. This score ranges from 4 (fully active patient with no performance limitations) to 0 (bedridden, completely disabled) [23].

The patients were assessed at baseline, at 3 and 6 weeks and then at 3, 6, 9, and 12 months after transplantation. In these exploratory analyses, we use clinical data and PROs at baseline.

### Statistical analyses

All descriptive statistics were performed and reported overall and by study group affiliation (intervention and control). Due to the exploratory nature of the current study, we assessed all the scales from the QLQ-C30 questionnaire without selecting any primary scales. For each scale, we used Cox proportional hazard models to investigate possible associations of the corresponding pre-treatment score with the risk of dying (for any cause) over 1 year, and logistic regression for 1-year NRM, respectively. For each clinical outcome, we first ran a univariable model using sociodemographic and clinical variables. Then, we ran a multivariable model, including the statistically significant variables in univariable analysis, i.e., age, living arrangements (living alone vs. not), stem cell source (peripheral blood vs. bone marrow), and EBMT score. In addition, we forced key variables that we deemed important from a clinical point of view into the multivariable model, i.e., gender, study group (intervention vs. control), HCT-CI score, body mass index and donor type (related vs. unrelated). Then, for each outcome, we ran the same multivariable model also including the pre-treatment score of each QLQ-C30 scale. The additional information provided by a scale was evaluated by the likelihood ratio test, testing the null hypothesis that the scale did not significantly increase the model fit when added to sociodemographic and clinical variables. Values are reported as hazard ratio (HR) or odds ratio (OR). The significance level was set at 0.05 with no adjustment for multiple testing, and all statistical tests were two-sided. All analyses were performed by SAS software v.4 (SAS Institute Inc., Cary, NC, USA).

## Results

### Patient inclusion

**Fig. 1:**
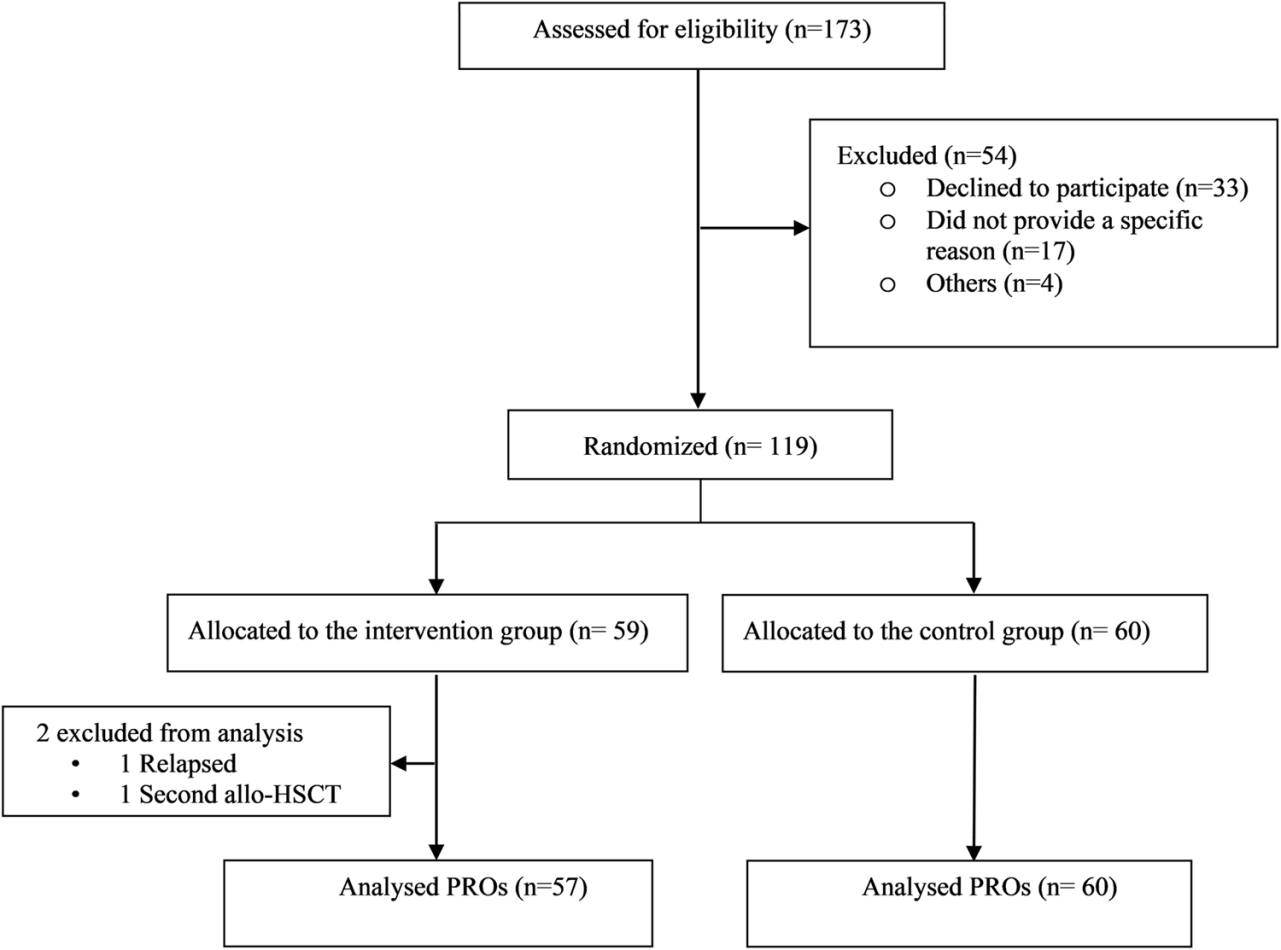
Flow chart showing the inclusion process of patients receiving allogeneic hematopoietic stem cell transplantation participating in the study, and the number of patient-reported outcomes that was analyzed

In the original RCT, 173 patients ≥ 18 years of age undergoing allo-HSCT with myeloablative conditioning were assessed for eligibility. Of these, 119 patients consented and were randomly assigned to receive the nutrition intervention or the standard total parental nutrition. Two patients in the intervention group were excluded from further analysis (Fig. 1), leaving 117 patients for the intention-to-treat analysis (intervention *n*=57, control *n*=60).

### Patient characteristics

Most patients (74%) were categorized as low risk according to the HCT-CI score, and acute myeloid leukemia was the predominant diagnosis Table 1. The different baseline characteristics were evenly distributed between the two study groups, indicating that the randomization was adequate. Thirty-five (30%) patients died during the first year, 15 patients of these due to relapse. There was no significant difference in 1-year survival between the two study groups. For descriptive purposes, the QoL baseline scores for all patients combined and per groups are shown in Table 2.

**Table 1:** Baseline characteristics

Characteristics	Intervention (*n*=57)	Control (*n*=60)	Total (*n*=117)
Median age in years (minimum-maximum)	45 (19-65)	41 (18-62)	(18-65)
Female	20 (35)	25 (42)	45 (38)
AML	36 (63)	31 (51)	67 (57)
ALL	6 (10)	10 (17)	16 (14)
CML	2 (4)	7 (12)	9 (8)
CMML	3 (5)	3 (5)	6 (5)
MDS	6 (11)	5 (8)	11 (9)
Other	4 (7)	4 (7)	8 (7)
Donor			
HLA-identical sibling	17 (30)	13 (22)	30 (26)
HLA-identical unrelated	40 (70)	47 (78)	87 (74)
Stem cell source			
Bone marrow	25 (44)	27 (45)	52 (44)
Peripheral blood	32 (56)	33 (55)	65 (56)
Sex mismatch	17 (30)	10 (17)	27 (23)
Conditioning			
Busulfan + cyclophosphamide	56 (98)	56 (93)	112 (96)
TBI + cyclophosphamide	1 (2)	4 (7)	5 (4)
HCT-CI risk groups			
Low risk	42 (74)	45 (75)	87 (74)
Intermediate risk	8 (14)	10 (17)	18 (15)
High risk	7 (12)	5 (8)	12 (10)
EBMT score			
0–3	33 (58)	36 (60)	69 (59)
4	14 (24)	14 (23)	28 (24)
5–7	10 (18)	10 (17)	20 (17)
Performance status ECOG			
0	55 (96)	54 (90)	109 (93)
1	2 (4)	6 (10)	8 (7)
Education			
Elementary school	4 (7)	7 (12)	11 (9)
High school	27 (47)	34 (57)	61 (52)
University/college	26 (46)	19 (32)	45 (38)
Home situation			
Alone	6 (11)	12(20)	18 (15)
With others	51 (89)	48 (80)	99 (85)

*AML* acute myeloid leukemia, *ALL* acute lymphocytic leukemia, *CML* chronic myeloid leukemia, *CMML* chronic myelomonocytic leukemia, *MDS* myelodysplastic syndrome, *CMV* cytomegalovirus, *TBI* total body irradiation, *HCT-CI* Hematopoietic Cell Transplantation Comorbidity Index, *EBMT* European Bone Marrow Transplantation risk score, *ECOG* Eastern Cooperative Oncology group

Values are number (%) unless otherwise stated

**Table 2:** Baseline quality of life scores

QOL-C30	Intervention	Control	Total
	(*n*=57)	(*n*=60)	(*n*=117)
Functional scales^1^			
Physical functioning (PF)	78 ± 16	79 ± 20	78 ± 18
Role functioning (RF)	50 ± 30	56 ± 36	53 ± 33
Emotional functioning (EF)	82 ± 18	83 ± 20	82 ± 19
Cognitive functioning (CF)	80 ± 22	83 ± 24	82 ± 23
Social functioning (SF)	50 ± 28	48 ± 30	49 ± 29
Global health status (QL)	68 ± 19	68 ± 23	68 ± 21
Symptom scales			
Fatigue	43 ± 23	39 ± 26	41 ± 24
Nausea/vomiting	6 ± 14	12 ± 18	9 ± 16
Pain	11 ± 18	10 ± 20	10 ± 19
Dyspnea	25 ± 26	24 ± 31	25 ± 29
Sleeping disturbance	26 ± 31	25 ± 31	26 ± 31
Appetite loss	18 ± 25	15 ± 23	17 ± 24
Constipation	11 ± 20	14 ± 25	12 ± 23
Diarrhea	16 ± 21	11 ± 20	13 ± 21
Financial impact	18 ± 27	16 ± 31	17 ± 29

^1^Higher scores on the functional and global QoL scales indicate better functioning, whereas higher scores on the symptom scales and single items indicate higher symptom burden

Values are given as mean ± SD

### Univariable and multivariable prognostic factor analysis of 1-year overall survival

Univariable analyses of 1-year OS are shown in Supplementary Tables 1 and 2 whereas the corresponding multivariable analyses are given in Table 3. Both analyses are exploratory, and hence, *p*-values are not adjusted for multiplicity meaning that statistical significance only suggests an association. The multivariable analysis identified only the HCT-CI score (HR=1.414; 95% CI, 1.137 to 1.759, *p*=0.002) and the EBMT score (HR=1.387; CI, 1.020 to 1.887, *p=*0.037) as statistically significant, with the HCT-CI score also being significant in the univariable analysis. Global QoL was significant in the univariable analysis (HR=0.975; 95% CI, 0.953 to 0.998, *p*=0.04), (Supplementary Table 2), but this was not the case in the multivariable analysis.

**Table 3:** Multivariable analyses for 1-year overall survival: Clinical and sociodemographic variables

Variables	Hazard ratio	95% Confidence interval		*P*-value
Male gender	0.926	0.444	1.930	0.836
Age	0.999	0.969	1.029	0.940
Living alone	2.005	0.840	4.787	0.117
Study group (ref=intervention)	1.082	0.540	2.170	0.824
HCT-CI score	1.414	1.137	1.759	0.002
EBMT score	1.387	1.020	1.887	0.037
BMI	1.063	0.979	1.154	0.148
Related donor type	1.739	0.759	3.982	0.191
Stem cell source (ref=peripheral)	1.186	0.550	2.561	0.663

*HCT-CI* Hematopoietic Cell Transplantation Comorbidity Index, *ref* reference, *EBMT* European Bone Marrow Transplantation risk score, *BMI* body mass index

### Univariable analyses for 1-year non-relapse mortality

Univariable analysis on the prognostic significance of the clinical-sociodemographic variables are shown in Supplementary Table 3. The statistically significant clinical-sociodemographic variables related to 1-year NRM included age (OR=1.043; 95% CI, 1.002 to 1.085, *p*=0.038), living alone (OR=4.210; 95% CI, 1.384 to 12.809, *p*=0.011), stem cell source (OR=3.918; 95% CI, 1.221 to 12.567, *p*=0.022), and EBMT score (OR=2.082; 95% CI, 1.364 to 3.178, *p*=0.001). Supplementary Table 4 shows the univariable analysis of the predictive significance of PROs. Global QoL (OR=0.975; 95% CI, 0.953 to 0.998, *p*=0.034) was the only statistically significant predictor of 1-year NRM.

### Multivariable analyses for 1-year non-relapse mortality

The exploratory multivariable model without PROs contained nine variables, of which four were identified as candidates for being prognostic for 1-year NRM: living alone (OR=7.980; 95% CI, 1.701 to 37.446, *p*=0.009) HCT-CI (OR=1.616; 95% CI, 1.092 to 2.392, *p*=0.016), EBMT risk score (OR=2.891; 95% CI, 1.493 to 5.599, *p*=0.002), and stem cell source (OR=4.609; 95% CI, 1.024 to 20.740, *p*=0.046) (Table 4). *P*-values were not adjusted for multiplicity. After having run this model by adding separately each scale of the QLQ-C30, only appetite loss (i.e., higher appetite loss) was statistically associated with a slightly lower chance of 1-year NRM (OR=0.95; 95% CI, 0.908 to 0.994, *p*=0.026) (Table 5). The global QoL scale did no longer remain significant here, as it was in the univariable analysis (data not shown).

**Table 4:** Multivariable analyses for 1-year non-relapse mortality: Clinical and sociodemographic variables

Variables	Odds ratio	95% Confidence interval		*P*-value
Male gender	0.488	0.127	1.885	0.298
Age	0.999	0.946	1.055	0.965
Living alone	7.980	1.701	37.446	0.009
Study group (ref=intervention)	0.724	0.214	2.454	0.605
HCT-CI score	1.616	1.092	2.392	0.016
EBMT score	2.891	1.493	5.599	0.002
BMI	1.112	0.943	1.312	0.207
Related donor type	1.134	0.228	5.633	0.878
Stem cell source (ref=peripheral)	4.609	1.024	20.740	0.046

*HCT-CI* Hematopoietic Cell Transplantation Comorbidity Index, *ref* reference, *EBMT* European Bone Marrow Transplantation risk score, *BMI* body mass index

**Table 5:** Multivariable analyses for 1-year non-relapse mortality including both clinical/sociodemographic variables and patient-reported appetite loss

Variables	Odds ratio	95% Confidence interval		*P*-value
Male gender	0.521	0.128	2.126	0.363
Age	0.969	0.910	1.031	0.320
Living alone	9.856	1.784	54.446	0.001
Study group (ref=intervention)	0.614	0.163	2.306	0.470
HCT-CI score	1.815	1.133	2.908	0.013
EBMT score	3.843	1.733	8.525	0.001
BMI	1.104	0.918	1.329	0.293
Related donor type	1.806	0.341	9.559	0.487
Stem cell source (ref=peripheral)	7.141	1.339	38.094	0.021
Patient-reported appetite loss (EORTC QLQ-C30)	0.950	0.908	0.994	0.026

*HCT-CI* Hematopoietic Cell Transplantation Comorbidity Index, *ref* reference, *EBMT* European Bone Marrow Transplantation risk score, *BMI* body mass index

## Discussion

Our findings suggest that both the HCT-CI- and the EBMT scores at baseline were prognostic for 1-year OS and for 1-year NRM. None of the PROs from the EORTC QLQ C30 questionnaire remained significant in the multivariable analysis on 1-year OS. Moreover, the analyses suggested that only appetite loss, living alone, and stem cell source could predict 1-year NRM, and appetite loss could be the only PRO with an independent prognostic value.

The HCT-CI and the EBMT risk scores as predictors of outcomes in allo-HSCT recipients have been examined in both prospective and retrospective multi-center studies [24–26]. However, the most common outcomes used in evaluation of the HCT-CI score and the EBMT score are 2 and 5 years NRM and OS, which is different from our study showing results after 1-year follow-up. In line with this, a review published in 2016 on risk assessment before allo-HSCT showed that only 3 of 43 studies examining the validity of the HCT-CI score or the EBMT risk score and had 1-year NRM and/or OS as an outcome [24]. Moreover, only one of these studies showed that the HCT-CI score predicted 1-year NRM and 1-year OS, and that was in pediatric patients [27]. Thus, our study further validates the use of HCT-CI risk score for allo-HSCT recipients by lending support to its predictive value also at 1-year NRM.

Interestingly, our analysis suggest that both stem cell source and living alone could predict 1-year NRM, but not 1-year OS, in the multivariable analyses. This finding can be explained by the causes of treatment-related morbidity and mortality, which is the main complication of allo-HSCT. For example, it is commonly reported that patients who receive stem cells from peripheral blood compared to stem cells from the bone marrow have an increased risk of GVHD [21], which is a major cause of treatment-related morbidity and mortality. This finding and its impact on OS is perhaps counteracted by the higher likelihood of relapse in a patient receiving stem cells from bone marrow [28, 29], and may explain why we found no significant association between stem cell source and OS. Bone marrow as the stem cell source could increase the likelihood of relapse and thus mortality, while peripheral blood conversely may increase likelihood of side effects like GVHD and increased mortality. However, our study has not documented prognostic value for relapse exclusively (only potentially for OS and NRM), so this issue warrants further study.

With regard to living alone as a suggested predictor for 1-year NRM, it can be assumed that for transplanted patients with one or more complications, survival may depend on whether the patients have a care person [30, 31]. This is in contrast to mortality related to relapse, which is likely not impacted by status of living alone, and may thus explain why this was not significant for OS. This finding also corresponds with previous research showing that lack of social connections is associated with poor health, and that socioeconomic status like living alone is highly correlated with greater risk of cancer- related mortality and all major cause of death [30–33].

Somewhat surprisingly, our analysis suggested that appetite loss could be associated with 1-year NRM, so that a higher appetite loss would indicate a lower 1-year NRM. Most previous research has reached the opposite conclusion [34–40]. For example, Efficace et al. found that higher patient-reported appetite loss was associated with shorter OS in women with metastatic breast cancer [8], while McKernan et al. and Fang et al. found that baseline appetite loss remained an independent significant prognostic factor for patients with gastro-esophageal cancer [41, 42]. Possibly a higher appetite loss at baseline could be explained by a pre-allo-HSCT treatment of sub-groups of patients based on their underlying disease which may have led to a lower NRM. In this way, appetite loss could be a proxy for the underlying disease or the pre-allo-HSCT treatment which is the cause of lower mortality. It is also possible that this finding is caused by a bias in the study design as patients included in this nutritional study could have a higher awareness on nutrition and appetite. Notwithstanding, caution should be exercised in the interpretation of this finding as the OR we observed (0.95) was small, the analysis exploratory, and because the study was not powered for sub-group analysis.

In contrast to previous studies, we did not find any significant predictors of either 1-year OS or 1-year NRM among the tested PROs derived from the QLQ C30 questionnaire (except for appetite loss as discussed above). This is in contrast with Hamilton and collaborators’ extensive study on PROs being predictive for survival, relapse, and NRM after allo-HSCT, where they found that physical well-being was prognostic for overall mortality [11]. However, at variance with our findings, Hamilton et al. did not find the HCT-CI score to be a predictor for NRM [11]. Moreover, our study further lends support to the HCT-CI- and the EBMT scores as prognostic for 1-year OS and for 1-year NRM. Although none of the PROs predicted 1-year OS and NRM, it may be that the QLQ-C30 is not sufficiently sensitive to detect the most relevant QoL aspects, and that other more specific PRO measurement tools might have provided different results.

Our study had some limitations. The exploratory nature of our analysis should be considered when interpreting the results, and the sample size was not large enough to conduct specific sub-analyses. Also, we studied a rather homogeneous patient population that may limit the generalizability of our findings. A strength of our study is the high-quality data obtained in the context of a robust RCT setting in a well-defined patient group.

In conclusion, whereas our analysis suggest that the two common risk tools (HCT-CI and EBMT risk scores) were predictive for 1-year OS and 1-year NRM, baseline PROs data had little or no associations with these outcomes in this specific setting.

## Supplementary information

Supplementary file 1 **Supplementary table 1**. Univariable analyses for 1-year overall survival: Clinical and sociodemographic variables, **Supplementary table 2**: Univariable analyses for 1-year overall survival: Patient-reported outcomes from the EORTC QLQ-C30, **Supplementary table 3**. Univariable analyses for 1-year non-relapse mortality: Clinical and sociodemographic variables, **Supplementary table 4**. Univariable analyses for 1-year non-relapse mortality: Patient-reported outcomes from the EORTC QLQ-C30.

## Data Availability

The data that supports the findings of this study are available from the corresponding author on reasonable request.
