# Supplementary material for: The prognostic value of patient-reported outcomes in allogeneic hematopoietic stem cell transplantation: exploratory analysis of a randomized nutrition intervention trial
Source: Ann Hematol. 2023 Mar 3;102(4):927–35. doi: 10.1007/s00277-023-05149-x (PMC9998318; doi:10.1007/s00277-023-05149-x)
Supplement: Supplementary file 1 — Supplementary table 1. Univariable analyses for 1-year overall survival: Clinical and sociodemographic variables, Supplementary table 2: Univariable analyses for 1-year overall survival: Patient-reported outcomes from the EORTC QLQ-C30, Supplementary table 3. Univariable analyses for 1-year non-relapse mortality: Clinical and sociodemographic variables, Supplementary table 4. Univariable analyses for 1-year non-relapse mortality: Patient-reported outcomes from the EORTC QLQ-C30. [file 277_2023_5149_MOESM1_ESM.pdf]

Annals of Hematology, Supplementary Information

The prognostic value of patient-reported outcomes in allogeneic hematopoietic stem cell transplantation: exploratory analysis of a randomized nutrition intervention trial

Anne Marte Gudmundstuen, Fabio Efficace, Geir Erland Tjønnfjord, Kristin Joan Skaarud, Francesco Cottone, Marianne Jensen Hjermstad and Per Ole Iversen

Correspondence: Anne Marte Gudmundstuen, Department of Haematology, Oslo University Hospital, P.O. Box 4950 Nydalen, NO-0424 OSLO, Norway. E-mail: a.m.gudmundstuen@medisin.uio.no

Supplementary table 1. Univariable analyses for 1-year overall survival: Clinical and sociodemographic variables

Variables	Hazard ratio	95% Confidence interval		*P*-value
Male gender	1.031	0.516	2.059	0.931
Age	1.009	0.984	1.036	0.480
Having children	0.727	0.364	1.452	0.366
Being married	0.0551	0.514	2.164	0.884
Living alone	1.702	0.741	3.909	0.210
High education	0.905	0.448	1.829	0.781
Working full-time	0.712	0.275	1.838	0.482
Group (ref=intervention)	1.103	0.563	2.160	0.776
ECOG (ref=1)	0.363	0.050	2.649	0.317
Related donor type	1.170	0.559	2.446	0.677
Stem-cell source (ref=peripheral)	1.452	0.727	2.901	0.290
Sex mismatch (ref=yes)	1.861	0.907	3.820	0.090
Patient CMV (ref=positive)	0.959	0.434	2.118	0.918
Donor CMV (ref=positive)	0.862	0.435	1.706	0.669
EBMT score	1.288	0.994	1.669	0.056
HCT-CI score	1.326	1.077	1.632	0.008
High risk	2.190	0.906	5.294	0.082
BMI	1.059	0.980	1.145	0.147

ECOG Eastern Cooperative Oncology group, ref reference, CMV cytomegalovirus, EBMT European Bone Marrow Transplantation risk score, HCT-CI Hematopoietic Cell Transplantation Comorbidity Index, BMI body mass index.

Supplementary table 2: Univariable analyses for 1-year overall survival: Patient-reported outcomes from the EORTC QLQ-C30

Variables	Hazard ratio	95% Confidence interval		*P*-value
**Physical functioning and Global QoL scales**				
Physical functioning	0.991	0.974	1.009	0.316
Role functioning	0.992	0.981	1.002	0.114
Cognitive functioning	1.006	0.990	1.022	0.456
Social functioning	0.992	0.980	1.004	0.190
Emotional functioning	1.012	0.992	1.032	0.248
Global Health Status/QoL	0.984	0.968	0.999	0.038
**Symptoms**				
Fatigue	1.007	0.994	1.021	0.285
Nausea/Vomiting	0.983	0.958	1.009	0.204
Pain	1.007	0.992	1.022	0.388
Dyspnea	1.008	0.997	1.019	0.166
Appetite loss	0.990	0.974	1.006	0.208
Sleep disturbances	0.999	0.989	1.010	0.915
Constipation	0.991	0.974	1.009	0.319
Diarrhea	0.990	0.972	1.009	0.316
Financial difficulties	1.002	0.991	1.013	0.676

Supplementary table 3. Univariable analyses for 1-year non-relapse mortality: Clinical and sociodemographic variables

**Variable**	**Odds ratio**	**95% Confidence interval**		***P*-value**
Male gender	0.925	0.346	2.474	0.877
Age	1.043	1.002	1.085	0.038
Having children	1.096	0.385	3.123	0.864
Being married	0.704	0.261	1.898	0.489
Living alone	4.210	1.384	12.809	0.011
High education	1.386	0.524	3.666	0.510
Working full-time	1.463	0.471	4.549	0.511
Group (ref=intervention)	0.835	0.318	2.196	0.715
ECOG (ref=1)	0.677	0.079	5.827	0.723
Related donor type	0.683	0.209	2.231	0.528
Stem-cell source (ref=peripheral)	3.918	1.221	12.567	0.022
Sex mismatch (ref=yes)	2.073	0.731	5.878	0.170
Patient CMV (ref=positive)	0.932	0.306	2.844	0.902
Donor CMV (ref=positive)	0.649	0.238	1.767	0.397
EBMT score	2.082	1.364	3.178	0.001
HCT-CI score	1.367	0.988	1.890	0.059
High risk	1.725	0.423	7.040	0.447
BMI	1.094	0.979	1.223	0.114

ECOG Eastern Cooperative Oncology group, ref reference, CMV cytomegalovirus, EBMT European Bone Marrow Transplantation risk score, HCT-CI Hematopoietic Cell Transplantation Comorbidity Index, BMI body mass index.

Supplementary table 4. Univariable analyses for 1-year non-relapse mortality: Patient-reported outcomes from the EORTC QLQ-C30

**EORTC QLQ-C30**	**Odds ratio**	**95% Confidence interval**		***P*-value**
**Physical functioning and global QoL scales**				
Physical functioning	0.988	0.963	1.013	0.344
Role functioning	0.996	0.981	1.010	0.568
Cognitive functioning	1.004	0.982	1.026	0.714
Social functioning	1.003	0.986	1.019	0.755
Emotional functioning	1.012	0.984	1.041	0.398
Global health Status/QoL	0.975	0.953	0.998	0.034
**Symptoms**				
Fatigue	1.010	0.991	1.030	0.296
Nausea/vomiting	0.967	0.924	1.011	0.138
Pain	1.015	0.993	1.038	0.176
Dyspnea	1.002	0.985	1.020	0.788
Appetite loss	0.970	0.941	1.000	0.050
Sleep disturbances	1.001	0.986	1.017	0.897
Constipation	0.989	0.964	1.015	0.398
Diarrhea	1.000	0.977	1.024	0.989
Financial difficulties	0.997	0.980	1.015	0.758
